# Identification of lncRNA Signature of Tumor-Infiltrating T Lymphocytes With Potential Implications for Prognosis and Chemotherapy of Head and Neck Squamous Cell Carcinoma

**DOI:** 10.3389/fphar.2021.795205

**Published:** 2022-02-15

**Authors:** Liping Wang, Gui Yang, Guohong Liu, Yunbao Pan

**Affiliations:** ^1^ Department of Laboratory Medicine, Zhongnan Hospital of Wuhan University, Wuhan University, Wuhan, China; ^2^ Department of Radiology, Zhongnan Hospital of Wuhan University, Wuhan University, Wuhan, China

**Keywords:** head and neck squamous cell carcinoma, tumor-infiltrating T lymphocytes, long noncoding RNAs, tumor microenvironment, immunotherapy

## Abstract

**Purpose:** We systematically analyzed HNSCC-infiltrating T lymphocytes lncRNAs (HILTlncRNAs) to assess their predictive value for the survival outcome and immunotherapy response of patients with anti-programmed death-1 (PD-1) therapy and to evaluate their predictive power to chemotherapeutic agents.

**Methods:** HNSCC transcriptome and clinical information was obtained from The Cancer Genome Atlas (TCGA) database. Immunocell microarray data were obtained from the Gene Expression Omnibus (GEO) database. T-cell-specific lncRNAs were identified by differential expression analysis. Prognostic paired HILTlncRNAs (PHILTlncRNAs) were filtered and modeled by univariate cox, lasso and multivariate cox regression analysis. To construct lncRNA-miRNA-mRNA competitive endogenous RNA (ceRNA) regulatory networks, differentially expressed mRNAs in HNSCC patients were incorporated, microRNAs and differentially expressed mRNAs interacting with T-cell-specific lncRNAs were filtered out based on miRcode, miRDB, miRTarBase, and TargetScan databases.

**Results:** 75 T-cell-specific lncRNAs and 9 prognostic PHILTlncRNAs were identified. Low-risk HNSCC patients had a better prognosis and significant immune cell infiltration, driving the immune response. Differential expression of RNA-binding proteins (RBPs), PD-1 and programmed cell death 1 ligand 1 (PD-L1) was demonstrated in the high and low risk groups of HNSCC patients. In the high risk group, high expression of PD-1 improved patient prognosis, whereas the opposite was observed in the low-risk group. The promoter methylation levels of two RBPs (DNMT1 and ZC3H12D) were decreased in HNSCC patients compared with normal samples, their expression levels were positively correlated with PD-1 and PD-L1 levels and T-cell infiltration. Finally, we screened the sensitivity of HNSCC patients to chemotherapeutic agents and found it differed between high and low risk groups.

**Conclusion:** HILTlncRNAs provided a theoretical basis for immune targeted therapy and drug development.

## Introduction

Head and neck squamous cell carcinoma (HNSCC) occurs mainly in the mucosal epithelium of the pharynx and oral cavity. Although its epidemiology has changed considerably in recent years, with both an incidence decline in cigarette smoking-associated HNSCC and an increase in human papillomavirus (HPV)-associated HNSCC, it remains a heavy burden on healthcare systems worldwide with 930,000 new cases of HNSCC and 470,000 HNSCC-related deaths each year (McDermott and Bowles, 2019; Johnson et al., 2020; Sung et al., 2021).

The tumor microenvironment (TME) consists of various cell types, including tumor cells, immune cells, endothelial cells, adipocytes and fibroblasts, and a variety of structures, such as blood vessels, lymphatic vessels and extracellular matrix, *etc*. The TME plays an important role in tumor growth, invasion, metastasis, diagnosis and treatment. High level of T lymphocyte infiltration in the TME or close to the tumor cell parenchyma may have prognostic value (Lei et al., 2016; Di Martino et al., 2019). High infiltration of CD4^+^ and CD8^+^ T cells in the TME has been associated with improved overall and relapse-free survival in patients with HNSCC, and could serve as an independent prognostic factor (Nguyen et al., 2016). Blockade of the T-cell immunoglobulin mucin 3 (TIM3) receptor reduces immunosuppression by downregulating regulatory T cells (Tregs) in HNSCC (Liu et al., 2018a). Elevated interleukin 23 (IL-23) and IL-6 levels released by HNSCC cells may promote T helper 17 (Th17) cell proliferation (Kesselring et al., 2010), while Th17 and Tregs proliferation is associated with the functional impairment of infiltrating CD8^+^ T cells in HNSCC (Kesselring et al., 2010; Liu et al., 2018b). Restoring exhausted T cells in the TME by blocking TIGIT/CD155 promotes antitumor immunity in HNSCC (Wu et al., 2019).

LncRNAs have been shown to play an important role in HNSCC prognosis. Cao et al. found that KTN1-AS1, LINC00460 and RP5-894A10.6 affected the survival of HNSCC patients (Cao et al., 2017). Wang et al. screened AC002066.1, AC013652.1 and AC016629.3 by cox regression analysis to construct a prognostic model for HNSCC. Three lncRNA co-expressed mRNAs were performed functional enrichment analysis. These mRNAs are involved in the regulation of angiogenesis, cell adhesion and extracellular matrix breakdown (Wang et al., 2018a). Diao et al. identified four lncRNAs including RP11-366H4.1, LINC01123, RP11-110I1.14 and CTD-2506J14.1 which are closely associated with overall survival of HNSCC patients (Diao et al., 2019). Zhang et al. constructed a risk value model for HNSCC based on 15 lncRNAs (FOXD2-AS1, MYOSLID, WFDC21P, AC073130.1, AL078644.1, LINC01234, AC243773.2, C5orf66-AS1, LINC02041, AC012213.4, LINC01305, AC108134.1, ALMS1-IT1, LINC02099 and AC019171.1), which can effectively predict overall survival and stratified patients (Zhang et al., 2019). In particular, lncRNA LINC00460 regulated autophagy of HNSCC cells through regulation of the microRNA (miRNA)-206/stanniocalcin-2 axis (Xue et al., 2019). LncRNA MIR31HG promoted HNSCC cell proliferation by regulating the cell cycle through HIF1A and p21 (Wang et al., 2018b). LncRNA MX1-215 negatively regulated immunosuppression in HNSCC by interrupting H3K27 acetylation (Ma et al., 2020). An increasing number of studies have reported that immune-related lncRNAs were associated with the diagnosis and prognosis of HNSCC patients (Chen et al., 2021; Yin et al., 2021). However, the function of lncRNAs in the regulation of infiltrating T lymphocytes in HNSCC was not clear, thus we investigated the lncRNA regulatory network of infiltrating T lymphocytes in HNSCC and its clinical significance.

We proposed prognostic paired HNSCC-infiltrating T lymphocytes lncRNAs (PHILTlncRNAs) as new biomarkers for HNSCC, performed a systematic analysis and developed a model of PHILTlncRNAs to guide T lymphocyte infiltration into the HNSCC TME. We also predicted PD-1 immunotherapy response and screened the sensitivity of HNSCC patients to chemotherapy agent. We first extracted T-cell-specific lncRNAs, and then identified paired lncRNAs in HNSCC-infiltrating T lymphocytes (PHILTlncRNAs) to develop models of prognostic, risk assessment, and clinical parameter analysis. Subsequently, we predicted miRNAs and mRNAs which interact with T-cell-specific lncRNAs. Subsequently, we performed functional enrichment analysis and constructed ceRNA networks by intersecting the predicted mRNAs with differentially expressed mRNAs in HNSCC. Based on the developed prognostic model, we performed immune infiltration and immune function gene set enrichment analysis (GSEA), and also investigated the expression, methylation and mutation status of DNMT1, ZC3H12D, PD-1 and PD-L1 in HNSCC, and their effect on T-lymphocyte infiltration. Eventually, drug sensitivity analysis was performed in HNSCC patients to provide a clinical reference for screening of effective drugs.

## Methods

### Acquisition of Prognostic PHILTlncRNAs and Model Development

Transcriptomic data and clinical information of HNSCC were downloaded from The Cancer Genome Atlas (TCGA) database. Microarray data for T cells (GSE5105) and other immune cells (GSE59237, GSE6863, GSE23371, GSE25320, GSE27838, GSE28698, GSE37750, GSE39889, GSE8059, GSE49910 and GSE42058) based on the Affymetrix platform were downloaded from the Gene Expression Omnibus (GEO) database. The data were annotated by gene transfer format files obtained from Ensembl (http://asia.ensembl.org) and lncRNAs were filtered. The data were corrected using methods of ComBat in sva R package and normalizeBetweenArrays in limma R package. The differential expression analysis of lncRNAs between T cells and other immune cells was performed using limma R package to identify T-cell-specific lncRNAs (|logFC|> 1, FDR< 0.05). The intersected lncRNAs of T-cell-specific lncRNAs with lncRNAs in HNSCC were filtered to obtain HILTlncRNAs. These HILTlncRNAs were cyclically paired to construct a 0-or-1 matrix, in which when HILTlncRNA A is expressed at a higher level than HILTlncRNA B, the value is 1, otherwise it is 0. The pairs of lncRNAs with a value of 0 or 1 were successfully paired when they accounted for 20–80% of the total samples, and these successfully paired HILTlncRNAs were designated as PHILTlncRNAs. The prognostic candidate PHILTlncRNAs were filtered by univariate Cox regression analysis using the survival R package (*p* < 0.01), further screened by Lasso regression analysis. Those screened PHILTlncRNAs were used to perform multivariate Cox regression analysis to filter out the most prognostic PHILTlncRNAs. These identified PHILTlncRNAs were used to construct risk models using the survival, survminer and glmnet R packages. The risk score formula was as follows: Risk score = PHILTlncRNA1*β(PHILTlncRNA1) + PHILTlncRNA2*β(PHILTlncRNA2) + PHILTlncRNAi*β(PHILTlncRNAi), where β represents the regression coefficient values. The ROC curves were plotted using Kaplan-Meier method in survivalROC R package. The maximum value of sum of specificity and sensitivity was used as the cutoff value. HNSCC patients with risk score greater than the cutoff value are in the high risk group and the opposite in the low risk group.

### Assessing the Prognostic Value of PHILTlncRNAs

To assess the effectiveness of PHILTlncRNAs to predict survival, we performed Kaplan-Meier survival analysis using log-rank tests in the survival and survminer R packages between high and low risk groups. We further explored the survival status of HNSCC patients with increasing risk scores. The distribution of clinical parameters including age, gender, grade, stage and TMN stage of HNSCC patients in high and low risk groups were plotted using chi-squared test in the ComplexHeatmap R package. The differential level of risk scores among patients with different gender, or grade, or TN staging were derived using the Wilcoxon test in limma and ggpubr R packages. Finally, we performed univariate and multivariate Cox regression analysis for clinical parameters of age, gender, grade, stage, and risk scores in predicting survival of HNSCC patients using the survival R package, and thus accessed the feasibility of our model.

### ceRNA Network of HNSCC-Infiltrating T Lymphocytes

We predicted the interactions between T-cell-specific lncRNAs and miRNAs using the miRcode database (http://www.mircode.org/), identified the target mRNAs of miRNAs using the miRDB (http://mirdb.org/), miRTarBase (http://miRTarBase.cuhk.edu.cn/) and TargetScan (http://www.targetscan.org) databases. Hub mRNAs were obtained by intersecting the identified mRNAs with the differentially expressed mRNAs of HNSCC patients (|logFC|> 2, FDR< 0.05) using the Wilcoxon test. Subsequently, the corresponding lncRNAs and miRNAs which interact with the hub mRNAs were screened using cytoscape software to construct the ceRNA network. To characterize the molecular pathways in which these hub mRNAs are involved, we performed Kyoto Encyclopedia of Genes and Genomes (KEGG) and Gene Ontology (GO) enrichment analysis. The above procedures were conducted with R packages including limma, org.Hs.eg.db and clusterProfiler.

### Analysis of Immune Cell Infiltration

We performed differential analysis of immune cell infiltration using limma, ggpubr R packages, and tumor immune estimation resource 2 (TIMER2: http://timer.cistrome.org/) database between high and low risk groups of HNSCC patients. Then, we evaluated the correlation of risk score with immune cell infiltration in HNSCC patients using limma, scales, ggplot2, ggtext R packages, and the TIMER2 database. We explored the differences in immune function between HNSCC patients at high and low risk, by performing GSEA with GSEA 4.0.1 software, after downloading the gmt format files of IMMUNE_RESPONSE (M19817) and IMMUNE_SYSTEM_PROCESS (M13664) gene sets from molecular signatures’ database (http://www.gsea-msigdb.org/gsea/msigdb/search.jsp).

### Examining the relationship among RNA-binding proteins (RBPs), PHILTlncRNAs, PD-1 and PD-L1 in HNSCC

We analyzed the differential expression of *PDCD1* (gene encoding PD-1), *CD274* (gene encoding PD-L1) and two RBPs (*DNMT1* and *ZC3H12D*) in high and low risk groups using limma and ggpubr R packages. Correlation analysis of the expression of genes encoding PD-1 and PD-L1 and HILTlncRNAs was conducted with ggplot2, ggpubr and ggExtra R packages. To investigate the effect of PD-1 or PD-L1 expression combined with PHILTlncRNAs on the survival of HNSCC patients, we performed Kaplan-Meier survival analysis using survivor and survminer R packages with log-rank tests. The correlation of DNMT1, ZC3H12D, PD-1 and PD-L1 expression were analyzed by “Correlation Analysis” with Pearson correlation coefficient using data from TCGA database in the GEPIA2 (Gene Expression Profiling Interactive Analysis 2: http://gepia2.cancer-pku.cn/#degenes) platform. We also analyzed the correlation between *DNMT1* and *ZC3H12D* expression and CD4^+^ and CD8^+^ T cell infiltration, respectively, using the TIMER2 website “immune-gene” model, with partial Spearman’s correlation. The relationship between PD-L1, DNMT1, PD-1 and ZC3H12D expression and clinical parameters of HNSCC patients was analyzed using the “immune-outcome” model. Finally, we evaluated promoter methylation differences between DNMT1 and ZC3H12D in HNSCC patients on the UALCAN database (http://ualcan.path.uab.edu/analysis-prot.html).

### Mutation and Drug Sensitivity Analysis

We submitted the “Head and Neck Squamous Cell Carcinoma (TCGA, PanCancer Atlas)”, DNMT1, ZC3H12D, PD-1 (PDCD1) and PD-L1 (CD274) data to the “Query by gene” at the cBioPortal (https://www.cbioportal.org/) website. We obtained the mutation types and their frequency in these four genes in the “oncoprint” mode and genetic mutation differences in the altered and unaltered groups in the “plot” mode. Groupings and copy number variant type distributions were obtained in the “Comparison/Survival” modes. Finally, we performed drug sensitivity analysis using the limma, ggpubr, pRRophetic and ggplot2 R packages in HNSCC patients from TCGA database.

## Results

### Acquisition of Prognostic PHILTlncRNAs and Model Development

**FIGURE 1:**
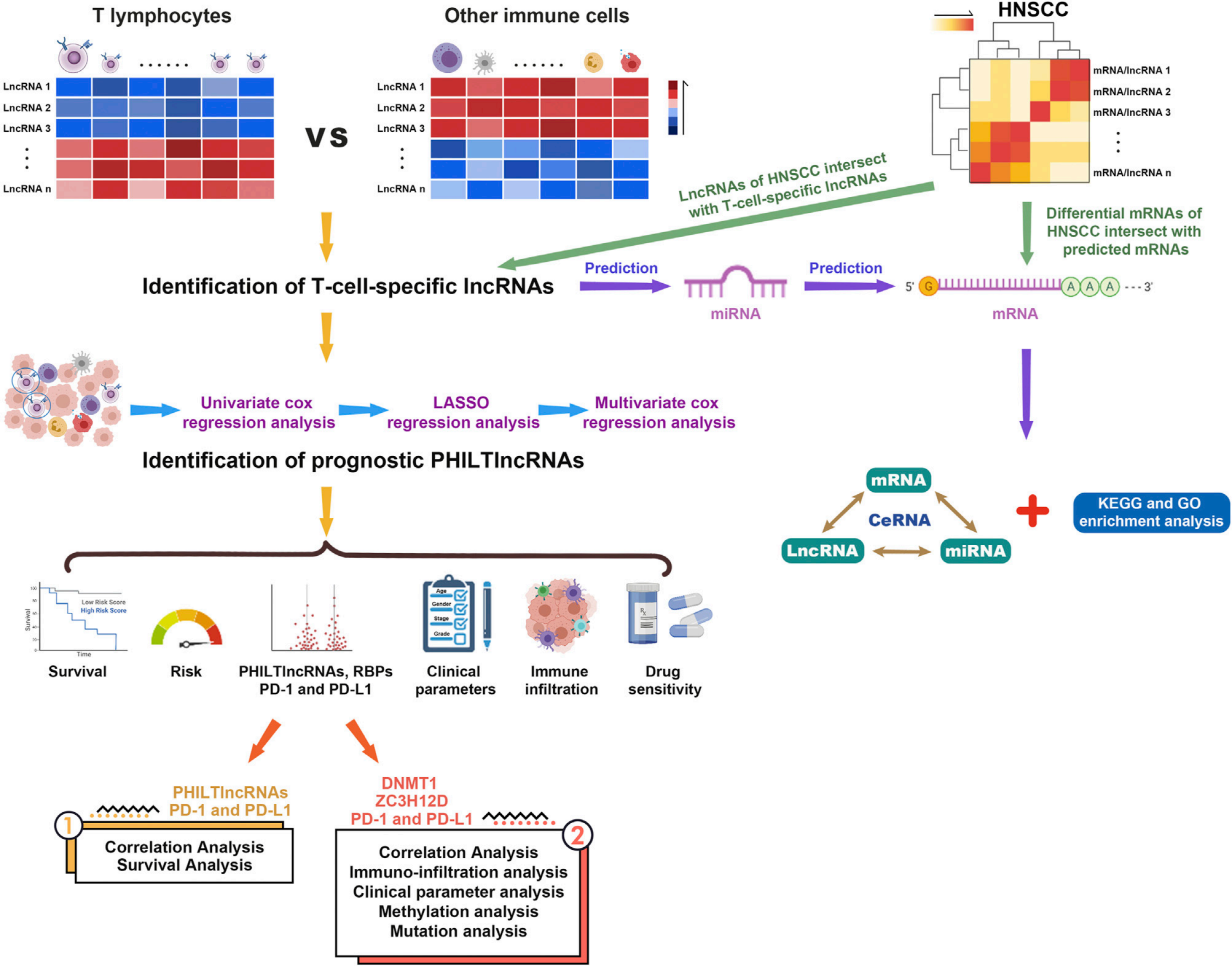
Flow chart of the entire analysis process.

**FIGURE 2:**
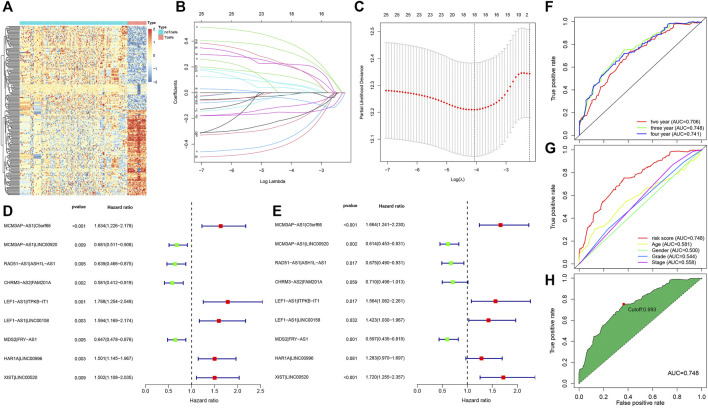
Acquisition of prognostic PHILTlncRNAs. **(A)** Differential expression analysis of lncRNAs in T cells and other immune cells from the GEO database. **(B)** Lasso regression modeling of 26 candidate prognostic PHILTlncRNAs obtained by univariate Cox regression analysis. **(C)** Optimal Log(λ) value obtained and used to filter 26 candidate prognostic PHILTlncRNAs. **(D)** Univariate Cox regression analysis of modeled PHILTlncRNAs. **(E)** Multivariate Cox regression analysis of modeled PHILTlncRNAs. **(F)** ROC curves for 2, 3 and 4 years to assess the model predictive value. **(G)** Comparison of the ROC curves of risk scores with the clinical parameters. **(H)** A maximum inflection point of 0.993 was obtained and used as the cut-off value to classify the high and low risk groups in this model.

A flow chart illustrating the steps of the whole analysis was shown in Figure 1. Primary sites of the samples from the TCGA database were demonstrated in Supplementary Table S1. After differential expression analysis of lncRNAs between T cells and other immune cells from the GEO database (Figure 2A), we predicted 75 T-cell-specific lncRNAs (|logFC|> 1, FDR< 0.05). Subsequently, the 75 T-cell-specific lncRNAs were intersected with lncRNAs of HNSCC and paired to obtain 848 PHILTlncRNAs. Through univariate Cox regression analysis (*p* < 0.01), we obtained 26 candidate prognostic PHILTlncRNAs, which were then screened by Lasso regression analysis for subsequent multivariate Cox regression analysis (Figures 2B,C). Ultimately, 9 prognostic PHILTlncRNAs were identified and modeled by multivariate Cox regression analysis (Figures 2D,E). Then, the predictive survival value of the model was assessed by plotting the ROC curves for 2, 3, and 4 years with area under curve (AUC) values of 0.706, 0.748, and 0.741, respectively, indicating that the model was successfully developed (Figure 2F). In addition, the AUC value of the 3-years ROC curve was significantly higher than those of other clinical parameters, indicating that the model developed using the risk score was reliable in predicting survival (Figure 2G). Our subsequent calculation of the AIC value for each point on the 3-years ROC curve revealed that the cutoff value to divide high and low risk groups was the maximum inflection point of 0.993 (Figure 2H).

### Evaluation of the Value of Prognostic PHILTlncRNAs

**FIGURE 3:**
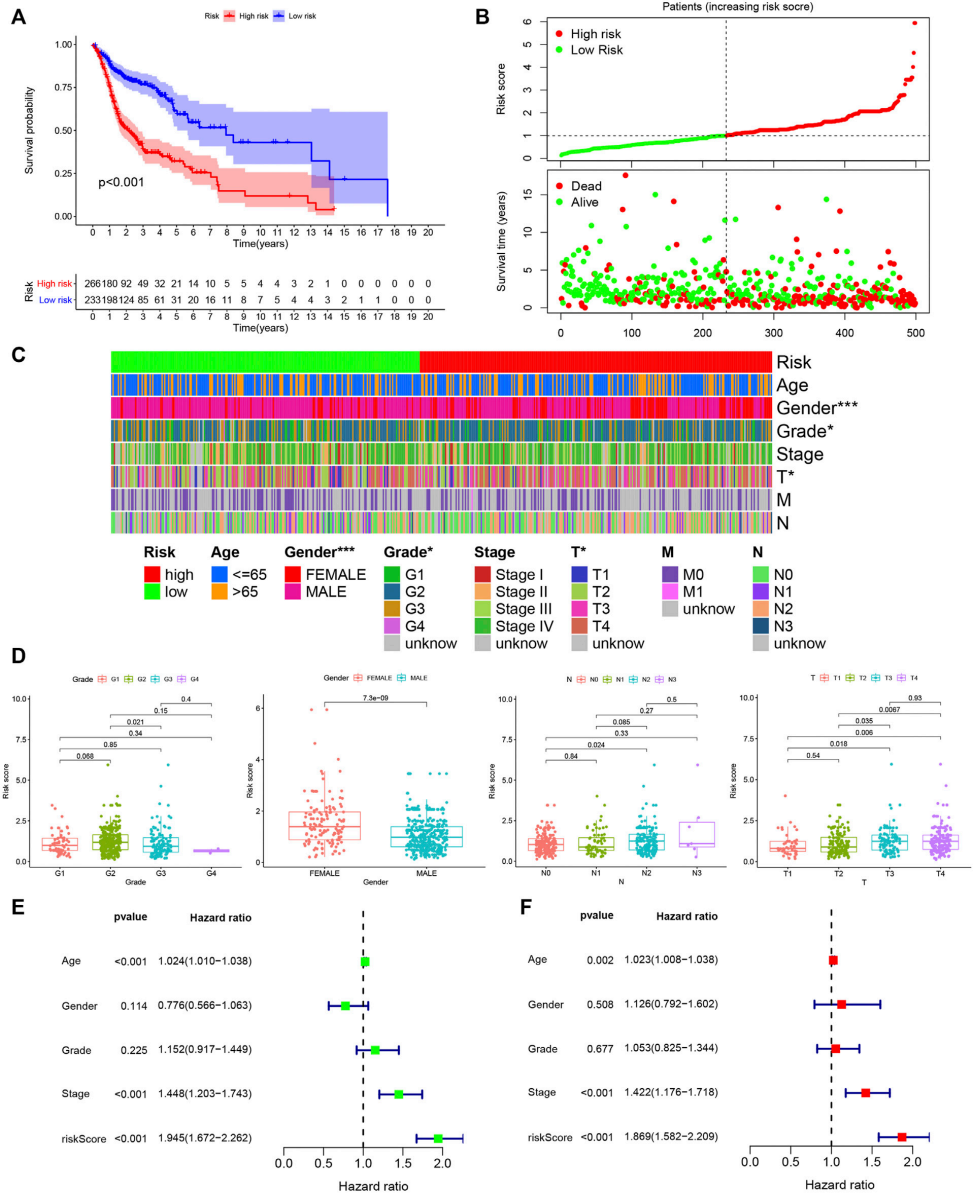
Assessing the prognostic value of PHILTlncRNAs. **(A)** Survival analysis of the high and low risk groups in HNSCC patients. **(B)** Risk score curve and survival status distribution of HNSCC patients. **(C)** Distribution of clinical parameters in high and low risk groups of HNSCC patients. **(D)** Differential risk score analysis among different clinical parameters in HNSCC patients. **(E)** Univariate Cox regression analysis of clinical parameters and hazard ratio. **(F)** Multivariate Cox regression analysis of clinical parameters and hazard ratio.

The prognosis of HNSCC patients in the high risk group was poor (*p* < 0.001), as shown in Figure 3A. Further, as the risk score increased, the number of deaths in the HNSCC group of patients increased (Figure 3B). The grade (*p* < 0.05), T-stage (*p* < 0.05) and sex (*p* < 0.001) of HNSCC patients differed in the high and low risk groups, with females predominating in the high risk group (Figure 3C). As shown in Figure 3D, the risk score was higher in G2 than in G3 (*p* = 0.021) stage, higher in female patients than in male patients (*p* = 7.3e-09), lower in N0 than in N2 (*p* = 0.024) stage, higher in T3 than in T1 (*p* = 0.018) stage, higher in T3 than in T2 (*p* = 0.035) stage, higher in T4 than in T1 (*p* = 0.006) and T2 (*p* = 0.0067) stages. In the univariate Cox regression analysis, age (HR = 1.024, 95% CI (1.010–1.038), *p* < 0.001), stage (HR = 1.448, 95% CI (1.203–1.743), *p* < 0.001) and risk score (HR = 1.945, 95% CI (1.672–2.262), *p* < 0.001) were associated with the prognosis of HNSCC patients (Figure 3E). In the multivariate Cox regression analysis, age (HR = 1.023, 95% CI (1.008–1.038), *p* = 0.002), stage (HR = 1.422, 95% CI (1.176–1.718), *p* < 0.001) and risk score (HR = 1.869, 95% CI (1.582–2.209), *p* < 0.001) were associated with the prognosis of HNSCC patients (Figure 3F). We further stratified the HNSCC patients by clinical parameters and performed multivariate cox regression analysis for each stratum of HNSCC patients and included lncRNA-miRNA-mRNA in the analysis. The results were shown in Supplementary Tables S2–S9.

### ceRNA Network of lncRNA-miRNA-mRNA in HNSCC-Infiltrating T Lymphocytes

**FIGURE 4:**
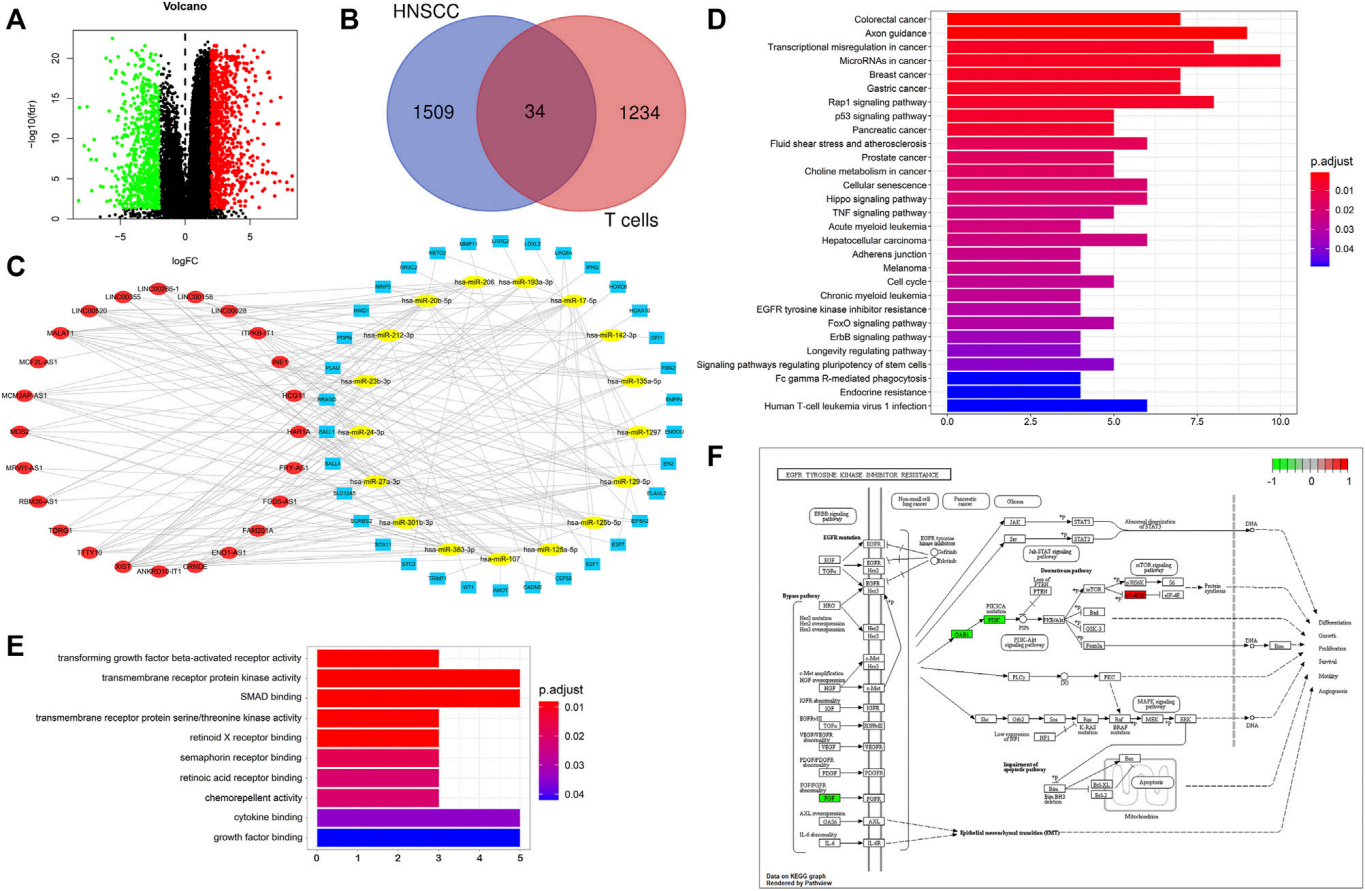
CeRNA network of T cells in HNSCC. **(A)** Differential mRNA expression analysis of HNSCC from TCGA database: |logFC|> 2, FDR< 0.05, upward adjustment in red and downward adjustment in green. **(B)** Venn diagram for hub mRNAs, blue: differential mRNAs of HNSCC, red: mRNAs predicted by miRNAs of T cells. **(C)** CeRNA network, blue: hub mRNAs, yellow: miRNAs, red: lncRNAs. **(D)** KEGG enrichment analysis of hub mRNAs. **(E)** GO enrichment analysis of hub mRNAs. **(F)** Hub mRNAs involved in the EGFR tyrosine kinase inhibitor resistance pathway mechanism regulation.

We performed differential expression analysis on the mRNA data of HNSCC from the TCGA database (|logFC|> 2, FDR< 0.05), and identified 1,543 differentially expressed mRNAs in HNSCC samples compared with normal samples (Figure 4A). We predicted 75 T-cell-specific lncRNAs that bind miRNAs using the miRcode database and identified 1,268 mRNAs that bind to these miRNAs using the miRDB, miRTarBase and TargetScan databases. We identified 34 hub mRNAs by intersecting the predicted mRNAs with the differential expressed mRNAs in HNSCC patients (Figure 4B). The 34 hub mRNAs were used to screen the corresponding lncRNAs and miRNAs, and a ceRNA network was constructed using the cytoscape software to visually establish their interconnections (Figure 4C). The KEGG enrichment analysis performed on these 34 hub mRNAs showed that they were involved in the regulation of the rap1 signaling pathway, p53 signaling pathway and EGFR tyrosine kinase inhibitor resistance, *etc* (Figure 4D). In addition, the GO enrichment analysis performed on these 34 hub mRNAs revealed that they were involved in various biological processes, including transforming growth factor beta-activated receptor activity, transmembrane receptor protein kinase activity and SMAD binding (Figure 4E). The hub mRNA was found to be involved in the mechanism of EGFR tyrosine kinase inhibitor resistance pathway, as shown in Figure 4F.

### Analysis of Immune Cell Infiltration

**FIGURE 5:**
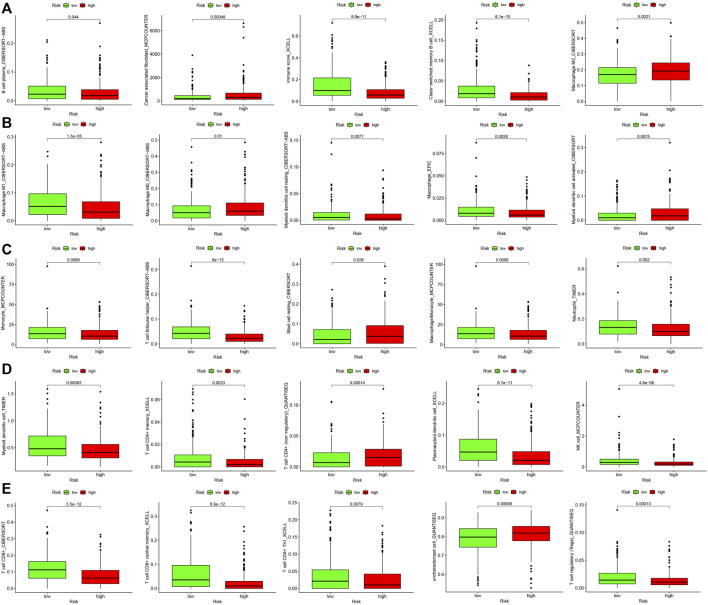
Differential analysis of immune cell infiltration in high- and low-risk groups. **(A)** B cell plasma, Cancer associated fibroblast, Immune score, Class-switched memory B cell and Macrophage M2. **(B)** Macrophage M1, Macrophage M0, Myeloid dendritic cell resting, Macrophage and Myeloid dendritic cell activated. **(C)** Monocyte, T cell follicular helper, Mast cell resting, Macrophage/Monocyte and Neutrophil. **(D)** Myeloid dendritic cell, T cell CD4^+^ memory, T cell CD4^+^ (non-regulatory), Plasmacytoid dendritic cell and NK cell. **(E)** T cell CD8^+^, T cell CD8^+^ central memory, T cell CD4^+^ Th1, Uncharacterized cell and T cell regulatory (Tregs).

**FIGURE 6:**
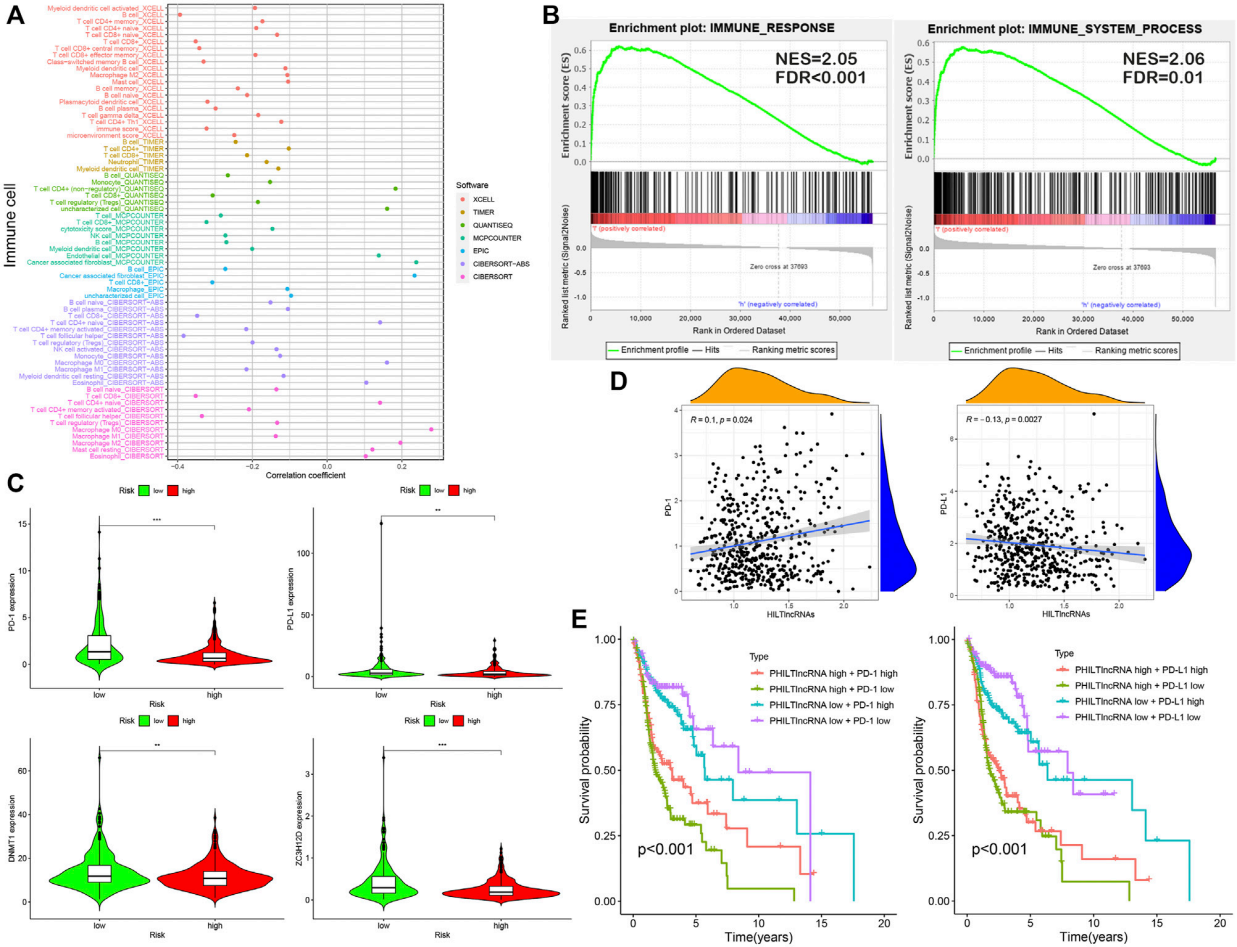
Analysis of immune infiltration and effect of PD-1/PD-L1 on patient prognosis. **(A)** Correlation between risk scores and immune cell infiltration in patients with HNSCC. **(B)** GSEA: immune response and immune system process. **(C)** Differential expression analysis with PD-1, PD-L1, DNMT1 and ZC3H12D. **(D)** Correlation analysis of PD-1, PD-L1 and HILTlncRNAs. **(E)** Survival analysis for four groups of HNSCC patients with PD-1, PD-L1 and PHILTlncRNAs.

The results of the differential analysis of immune cell infiltration in the high and low risk groups of HNSCC patients were shown in Figure 5, which showed that the extent of the immune cell infiltration decreased with increasing risk scores of HNSCC patients (Figure 6A). Our findings, by GSEA enrichment analysis, that the gene sets of IMMUNE_RESPONSE and IMMUNE_SYSTEM_PROCESS were both highly expressed in the low risk group and the gene sets in the low risk group were enriched in immune response (NES = 2.05, FDR< 0.001) and immune system process (NES = 2.06, FDR = 0.01) further verified the differences in immune function between the high and low risk groups, and also implied that the immune response and immune system process were biologically active in the low risk group of HNSCC patients (Figure 6B).

### Examining the Relationship Among RBPs, PHILTlncRNAs, PD-1 and PD-L1 in HNSCC

**FIGURE 7:**
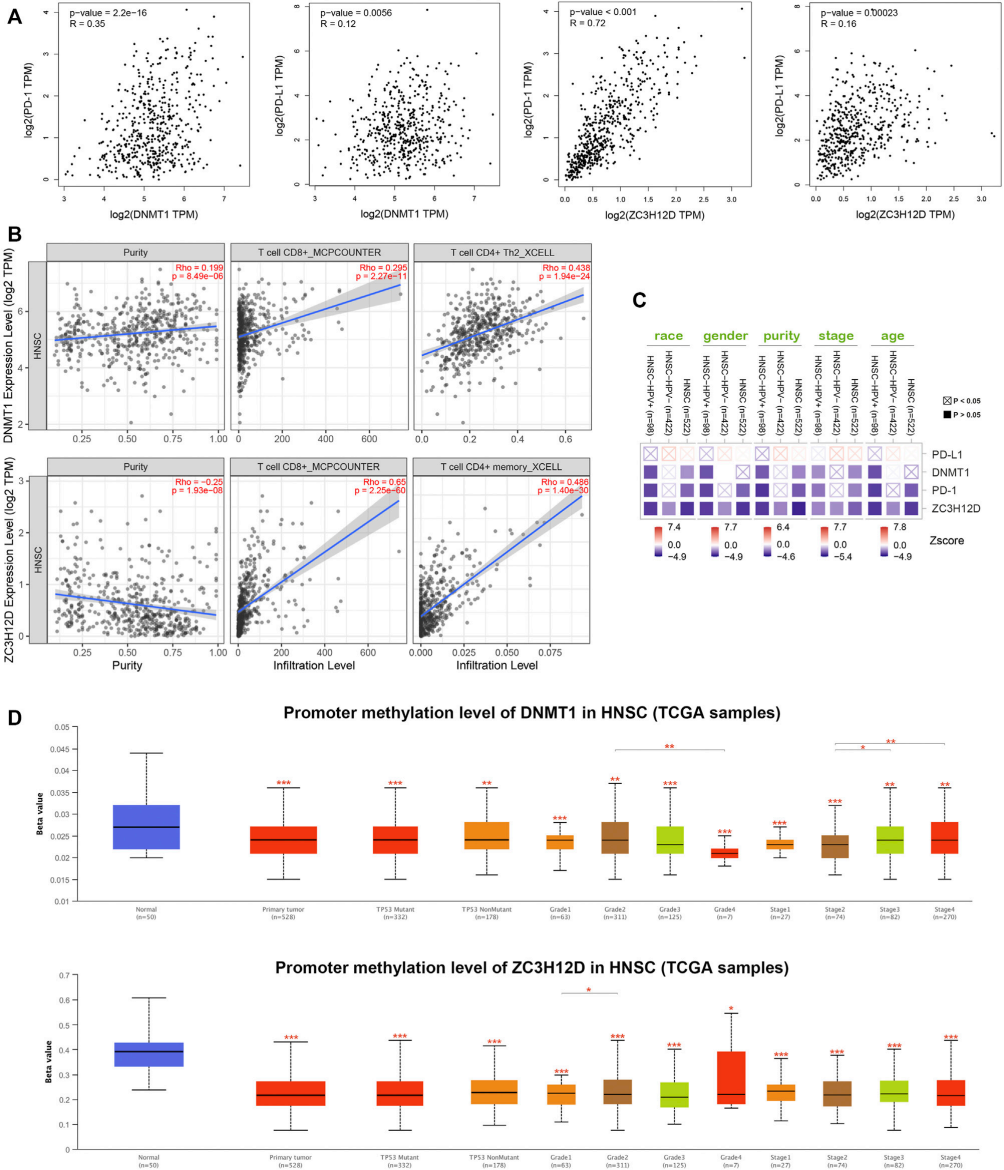
DNMT1 and ZC3H12D comprehensive analysis in HNSCC. **(A)** Correlation analysis of PD-1, PD-L1, DNMT1 and ZC3H12D. **(B)** DNMT1 and ZC3H12D correlated with CD4^+^ and CD8^+^ T cells infiltration. **(C)** Expression of PD-L1, DNMT1, PD-1 and ZC3H12D associated with clinical parameters in HNSCC patients. **(D)** Promoter methylation differences between DNMT1 and ZC3H12D, *p < 0.05, **p < 0.01, ***p < 0.001.

PD-1 and PD-L1 are immunotherapeutic targets commonly used in clinical practice; While RBPs, DNMT1 and ZC3H12D, are closely associated with tumors progression (Gerstberger et al., 2014; Du et al., 2018; Jiang et al., 2019; Zhu et al., 2021). In this study, we found that the genes encoding PD-1 and PD-L1, DNMT1 and ZC3H12D were highly expressed in the low risk group of HNSCC patients (Figure 6C). In addition, the HILTlncRNAs were found to be positively correlated with the expression of the gene encoding PD-1 (R = 0.1 and *p* = 0.024), while HILTlncRNAs were found to be negatively correlated with the expression of the gene encoding PD-L1 (R = −0.13 and *p* = 0.0027) (Figure 6D). As shown in Figure 6E, we divided the HNSCC patients into four groups, and HNSCC patients in the low risk group based on PHILTlncRNAs and PD-1 low expression had the best prognosis compared with the other three groups, while the HNSCC patients in high risk group and PD-1 low expression had the worst prognosis compared with the other three groups. The survival rate of HNSCC patients with high PD-1 expression in the low risk group was lower than that in the other groups, while high PD-1 expression in the high risk group was beneficial to improve the survival rate of HNSCC patients (*p* < 0.001). It was revealed similar effects as those of PHILTlncRNAs and PD-L1 on the survival of HNSCC patients (*p* < 0.001). To investigate whether the two RBPs (DNMT1 and ZC3H12D) were related to PD-1 and PD-L1, we performed correlation analysis and found that DNMT1 (*p* = 2.2e-16 and R = 0.35) and ZC3H12D (*p* < 0.001 and R = 0.72) were positively correlated with the expression of the gene encoding PD-1. DNMT1 (*p* = 0.0056 and R = 0.12) and ZC3H12D (*p* = 0.00023 and R = 0.16) were both positively correlated with the gene encoding PD-L1 (Figure 7A). DNMT1 was positively correlated with CD8^+^ T cell (R = 0.295, *p* = 2.27e-11 and MCPCOUNTER algorithm) and CD4^+^ Th2 cell (R = 0.438, *p* = 1.94e-24 and XCELL algorithm) infiltration. ZC3H12D was positively correlated with CD8^+^ T cell (R = 0.65, *p* = 2.25e-60 and MCPCOUNTER algorithm) and CD4^+^ memory cell (R = 0.486, *p* = 1.40e-30 and XCELL algorithm) infiltration (Figure 7B). We also found that the expression of both DNMT1 and gene encoding PD-1 in HNSCC HPV + patients was negatively correlated with clinical parameters (race, sex, purity, stage and age) (*p* < 0.05). The expression of ZC3H12D in HNSCC, HNSCC HPV+ and HNSCC HPV- patients (*p* < 0.05) was negatively correlated with clinical parameters (Figure 7C). We also found that the promoter methylation level of DNMT1 was differentially expressed in HNSCC and normal samples, Grade 2 and Grade 4, and in stage 2, 3 and 4. Additionally, the promoter methylation level of ZC3H12D was differentially expressed in HNSCC and normal individuals, and in Grade 1 and Grade 2 (Figure 7D).

### Mutation and Drug Sensitivity Analysis

**FIGURE 8:**
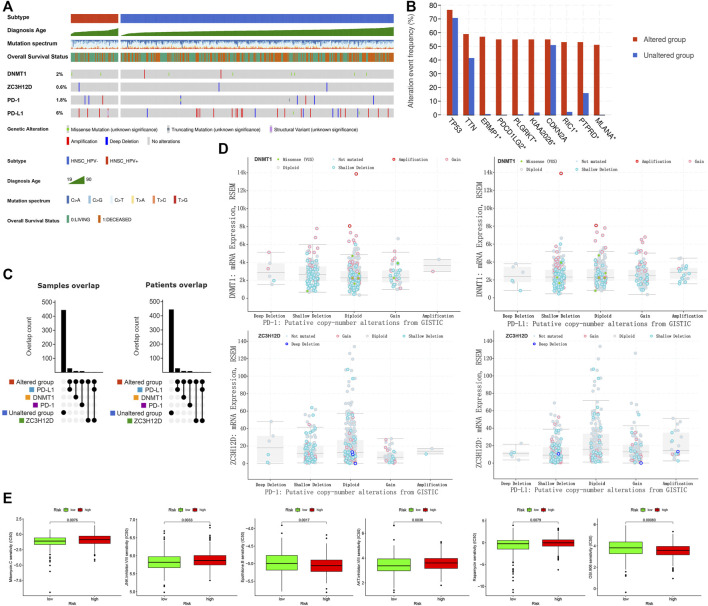
Mutation and drug sensitivity analysis in HNSCC patients. **(A)** Mutation types and frequency distribution in *DNMT1*, *ZC3H12D*, PD-1 and PD-L1. **(B)** Genetic mutation differences in altered and unaltered groups. **(C)** Grouping and overlap. **(D)** Copy number variant distribution of *DNMT1*, *ZC3H12D*, PD-1 and PD-L1. **(E)** Drug sensitivity analysis of high and low risk groups in HNSCC patients.

The mutations in *DNMT1*, *ZC3H12D*, PD-1 (*PDCD1*) and PD-L1 (*CD274*) detected in HNSCC patients were shown in Figure 8A. We included *DNMT1*, *ZC3H12D*, PD-1 (*PDCD1*) and PD-L1 (*CD274*) into the mutation classification. HNSCC patients were divided into the mutated group as long as one gene in the patient was mutated. *ERMP1*, *PDCD1LG2*, *PLGRKT*, *KIAA 2026*, *RIC1*, *PTPRD* and *MLANA* (*p* < 0.001) were mutated at high frequency in the mutant group (Figure 8B). Subsequently, after further refining the grouping into the altered, PD-L1, DNMT1, PD-1, unaltered and ZC3H12D groups, we observed that one HNSCC patient had simultaneous mutations in the gene encoding PD-L1 and *ZC3H12D* (Figure 8C). Figure 8D showed DNMT1 mRNA expression and ZC3H12D mRNA expression among the copy number variant types of PD-1, PD-L1. Finally, we analyzed the response of HNSCC to chemotherapeutic agents. HNSCC patients in the low risk group were more sensitive to mitomycin C (*p* = 0.0076), JNK inhibitor VIII (*p* = 0.0033), AKT inhibitor VIII (*p* = 0.0038) and rapamycin (*p* = 0.0079), while less sensitive to epothilone B (*p* = 0.0017) and OSI 906 (*p* = 0.00083) compared with the patients in the high risk group, as can be concluded from the half maximal inhibitory concentration (IC_50_) (Figure 8E).

## Discussion

The metastasis and recurrence of HNSCC may be attributed to the abnormal interaction of immune cells with tumor cells and stromal cells in the TME (Zhang et al., 2020). Infiltrating cells in HNSCC were often divided into two types, one promoted tumor growth and the other inhibited it. The abundant tumor-associated macrophages (TAMs) infiltration in HNSCC was often accompanied by poor prognosis and lymphatic metastasis (Ni et al., 2015; Weber et al., 2016). Macrophages/monocytes in the TME regulated HNSCC stem cells through cluster of differentiation 44 (CD44) (Gomez et al., 2020). Serum IL-6, secreted by monocytes, was a predictor of relapse and survival in HNSCC patients (Kross et al., 2008). Exosomes secreted by HNSCC cells were aggregated in the TME and promoted endothelial cells to angiogenesis (Ludwig et al., 2018). Macrophages also secreted vascular endothelial growth factor (VEGF) to promote neovascularization (Sun et al., 2018). Significantly increased Tregs in the HNNSCC TME was frequently accompanied by recurrence (Weed et al., 2013; Stasikowska-Kanicka et al., 2018). Myeloid-derived suppressor cells (MDSCs) reduced cysteine levels and produced arginine to suppress T-lymphocyte activation (Parker et al., 2015), they also inhibited natural killer (NK) cell activity (Greene et al., 2020). Cancer-associated fibroblasts (CAFs) promoted T lymphocyte apoptosis, Tregs proliferation, and suppressed antitumor immunity in the TME (Takahashi et al., 2015). NK cells, CD8^+^ T cells, T helper 1 (Th1) cells, dendritic cells (DCs), M1 TAMs and N1 tumor-associated neutrophils (TANs) often played an antitumor role in the TME (Peltanova et al., 2019; Chen et al., 2020; Elmusrati et al., 2021). NK cells secreted immune interferon γ (IFN-γ), which induced infiltration of Th1 cells and MDSCs, and triggered adaptive immunity (Morvan and Lanier, 2016). NK cells recognized major histocompatibility complex class I (MHC-I) on the surface of tumor cells and secreted perforin and granzyme B to induce tumor cell death (Morvan and Lanier, 2016; Santos et al., 2019). TANs triggered CD8^+^ T lymphocyte proliferation and cancer cell apoptosis (Moses and Brandau, 2016; Michaeli et al., 2017). Tumor-infiltrating CD8 T+ cells can either produce granzymes or perforin to directly kill tumor cells or produce IFN-γ and tumor necrosis factor (TNF) to mediate cytotoxic antitumor immune responses (Hadrup et al., 2013). However, the precise classification of infiltrating immune cells in HNSCC needs to be further explored. The biomarkers in the TME and infiltrating immune cell phenotype may change in the malignant progression of the tumor, and the staging of a HNSCC based on these changes need to be investigated. The molecular mechanisms underlying the invasive edge of HNSCC also need to be studied in depth. Although lncRNAs are not translated into proteins, they can regulate tumor biology by affecting miRNA and mRNAs. LncRNAs played an important role in the TME through transducing signaling (Botti et al., 2019). For instance, lncRNA CamK-A activated the CaMK-NF-κB axis involved in TME remodeling (Sang et al., 2018). Besides, lncRNAs were involved in tumor-stroma crosstalk, and thus can be used as potential tumor biomarkers (Zhou et al., 2020).

Infiltrating T lymphocytes in the TME affected HNSCC progression. However, there were few studies focusing on lncRNA regulatory networks in HNSCC-infiltrating T lymphocytes. The role of lncRNAs in infiltrating T lymphocytes in the HNSCC TME remains to be elucidated. Multiple studies have also revealed that lncRNAs were associated with HNSCC patient prognosis. Chen *et al.* screened seven prognostic immune-related lncRNAs by univariate and multivariate Cox regression analysis, and classified HNSCC patients into high and low risk groups based on these lncRNAs. Low-risk HNSCC patients have a better prognosis, with large infiltrations of immune cells. In their study, a dataset of the identified immune-related genes was obtained from the ImmPort database and used to identify immune related lncRNAs through a co-expression strategy. Those lncRNAs were defined as immune related lncRNAs (cor >0.4) (Chen et al., 2021). In the current study, we optimized our method to screen tumor-infiltrating T lymphocytes specific lncRNAs. Candidate prognostic lncRNAs were screened by univariate Cox regression analysis, followed by Lasso regression screening, and ultimately by multivariate Cox regression analysis, which improved the reliability of the results. We also classified the HNSCC patients into high and low risk groups based on the cutoff values on ROC curves instead of using median values. Several studies identified immune-related lncRNA pairs that could well predict HNSCC patient prognosis (Mao et al., 2020; Yin et al., 2021). However, the role of specific lncRNAs in infiltrating T lymphocytes in HNSCC remains unclear. In the current study, we identified 9 prognostic PHILTlncRNAs and further studied their role in HNSCC. We found the expression of the two RBPs, DNMT1 and ZC3H12D, were positive associated with PD-1, PD-L1 expression. In agreement with our results, Liu et al. verified that DNMT1 positively correlated with PD-L1 expression in hepatocellular carcinoma (Liu et al., 2017). Yan et al. found that overexpression of DNMT1 resulted in an increase of PD-L1 in small cell lung cancer (SCLC) cells (Yan et al., 2016). However, the relationship between ZC3H12D and PD-1/PD-L1 has not been reported and need further verification in the future.

In conclusion, this study systematically analyzed HILTlncRNAs and proposed HILTlncRNAs as novel biomarkers to provide a theoretical basis for the research in the TME of HNSCC and to provide new ideas for clinical diagnosis, immune-targeted therapy and drug discovery.

## Data Availability

The original contributions presented in the study are included in the article. Further inquiries can be directed to the corresponding authors.
