# Supplementary material for: Identification of lncRNA Signature of Tumor-Infiltrating T Lymphocytes With Potential Implications for Prognosis and Chemotherapy of Head and Neck Squamous Cell Carcinoma
Source: Front Pharmacol. 2022 Feb 15;12:795205. doi: 10.3389/fphar.2021.795205 (PMC8886158; doi:10.3389/fphar.2021.795205)
Supplement: Supplementary file 1 [file Table1.DOCX]

**Table S1. Primary sites of the samples (n=546)**

| Types | Number (%) |
| --- | --- |
| Other and unspecified parts of tongue | 140 (25.64%) |
| Larynx | 123 (22.53%) |
| Other and ill-defined sites in lip, oral cavity and pharynx | 83 (15.20%) |
| Floor of mouth | 57 (10.44%) |
| Other and unspecified parts of mouth | 41 (7.51%) |
| Tonsil | 41 (7.51%) |
| Base of tongue | 22 (4.03%) |
| Gum | 11 (2.01%) |
| Oropharynx | 10 (1.83%) |
| Hypopharynx | 9 (1.65%) |
| Palate | 5 (0.92%) |
| Lip | 3 (0.55%) |
| Bones, joints and articular cartilage of other and unspecified sites | 1 (0.18%) |
