# Supplementary material for: Identification of lncRNA Signature of Tumor-Infiltrating T Lymphocytes With Potential Implications for Prognosis and Chemotherapy of Head and Neck Squamous Cell Carcinoma
Source: Front Pharmacol. 2022 Feb 15;12:795205. doi: 10.3389/fphar.2021.795205 (PMC8886158; doi:10.3389/fphar.2021.795205)
Supplement: Supplementary file 2 [file Table8.DOCX]

| Table S8. Multivariate cox regression analysis of CeRNA network genes in HNSCC patients (T1-T2) | | | | | | | | | | |  |
| --- | --- | --- | --- | --- | --- | --- | --- | --- | --- | --- | --- |
|  |  |  |  |  |  |  |  |  |  |  |  |
| Genes | | Coef | | HR | | HR.95L | | HR.95H | | P value |  |
| SALL4 | 1.087961773 | | 2.968218001 | | 1.014049784 | | 8.688250062 | | 0.047097203 | |  |
| MDS2 | 2.914475961 | | 18.43914705 | | 1.80803573 | | 188.0505667 | | 0.013900872 | |  |
| EN2 | -0.294845306 | | 0.744646771 | | 0.540829393 | | 1.025274921 | | 0.070763956 | |  |
| XIST | 0.344768449 | | 1.41166301 | | 1.062598075 | | 1.875396256 | | 0.017363794 | |  |
| MRVI1-AS1 | -5.62551574 | | 0.003604704 | | 0.000104799 | | 0.123988484 | | 0.001830486 | |  |
| RBM26-AS1 | 3.404014751 | | 30.08464025 | | 5.286049809 | | 171.2215381 | | 0.000124716 | |  |
| ITPKB-IT1 | -14.2577762 | | 6.43E-07 | | 1.26E-11 | | 0.032740213 | | 0.009930099 | |  |
| MMP11 | 0.014023773 | | 1.014122568 | | 1.004287256 | | 1.0240542 | | 0.004797348 | |  |
| TRIM71 | -45.9370879 | | 1.12E-20 | | 4.83E-34 | | 2.60E-07 | | 0.003438007 | |  |
| SOX11 | -2.639572214 | | 0.071391803 | | 0.011068143 | | 0.460491853 | | 0.005515116 | |  |
| SLC12A5 | 0.68977231 | | 1.993261636 | | 1.008928643 | | 3.937931563 | | 0.047083139 | |  |
| FGD5-AS1 | -0.10293746 | | 0.902183394 | | 0.853961778 | | 0.953127995 | | 0.000239878 | |  |
| HOXC6 | 0.472718677 | | 1.604349978 | | 1.089153948 | | 2.36324613 | | 0.016751164 | |  |
| hsa-miR-206 | 0.000108749 | | 1.000108755 | | 1.000060082 | | 1.00015743 | | 1.19E-05 | |  |
| hsa-miR-212-3p | 0.083469824 | | 1.087052412 | | 1.040865372 | | 1.135288941 | | 0.000164542 | |  |
| hsa-miR-363-3p | 0.016990547 | | 1.017135707 | | 1.00453009 | | 1.029899509 | | 0.007577654 | |  |
| INE1 | -1.208640847 | | 0.298602851 | | 0.096840921 | | 0.920722994 | | 0.035402136 | |  |
| LINC00158 | -13.67718699 | | 1.15E-06 | | 4.34E-11 | | 0.030360415 | | 0.008473024 | |  |
| CRNDE | 0.156434815 | | 1.169334536 | | 0.999534332 | | 1.367980283 | | 0.050683999 | |  |
