# Supplementary material for: Identification of lncRNA Signature of Tumor-Infiltrating T Lymphocytes With Potential Implications for Prognosis and Chemotherapy of Head and Neck Squamous Cell Carcinoma
Source: Front Pharmacol. 2022 Feb 15;12:795205. doi: 10.3389/fphar.2021.795205 (PMC8886158; doi:10.3389/fphar.2021.795205)
Supplement: Supplementary file 3 [file Table7.DOCX]

| Table S7. Multivariate cox regression analysis of CeRNA network genes in HNSCC patients (G3-G4) | | | | | | | | | | |  |
| --- | --- | --- | --- | --- | --- | --- | --- | --- | --- | --- | --- |
|  |  |  |  |  |  |  |  |  |  |  |  |
| Genes | | Coef | | HR | | HR.95L | | HR.95H | P value | |  |
| ANKRD10-IT1 | 1.224846265 | | 3.403642782 | | 1.956433741 | | 5.921378243 | | | 1.45E-05 |  |
| NETO2 | -0.175332841 | | 0.839177662 | | 0.710958393 | | 0.990520901 | | | 0.038214411 |  |
| E2F1 | -0.382867153 | | 0.681903482 | | 0.580251233 | | 0.801363844 | | | 3.34E-06 |  |
| AMOT | 1.711438341 | | 5.536919732 | | 2.806591833 | | 10.92338393 | | | 7.94E-07 |  |
| SALL4 | -7.926907933 | | 0.000360901 | | 1.76E-05 | | 0.007391433 | | | 2.67E-07 |  |
| FRY-AS1 | -114.1764474 | | 2.59E-50 | | 3.15E-67 | | 2.14E-33 | | | 9.18E-09 |  |
| MALAT1 | -0.34578148 | | 0.70766711 | | 0.600970111 | | 0.833307229 | | | 3.37E-05 |  |
| MCM3AP-AS1 | 15.38245832 | | 4791999.511 | | 3739.244466 | | 6141149508 | | | 2.52E-05 |  |
| TDRG1 | -0.270796237 | | 0.762771906 | | 0.628645032 | | 0.925515911 | | | 0.006061676 |  |
| WT1 | 3.140764085 | | 23.12152693 | | 1.903786124 | | 280.8114843 | | | 0.013687886 |  |
| MDS2 | 56.27336455 | | 2.75E+24 | | 1.6397E+16 | | 4.61E+32 | | | 5.74E-09 |  |
| EN2 | 0.577253906 | | 1.781140529 | | 1.303869329 | | 2.43311313 | | | 0.000286479 |  |
| SALL1 | 0.64811753 | | 1.911938274 | | 0.955716753 | | 3.82488635 | | | 0.066960519 |  |
| HOXA10 | 1.971270234 | | 7.179790716 | | 3.145720066 | | 16.38715259 | | | 2.84E-06 |  |
| ELAVL2 | 1.458390136 | | 4.299033096 | | 2.403735922 | | 7.688733769 | | | 8.80E-07 |  |
| PDPN | -0.021698493 | | 0.978535226 | | 0.953735434 | | 1.003979881 | | | 0.097580238 |  |
| CEP55 | 0.085190248 | | 1.088924213 | | 0.983386943 | | 1.205787761 | | | 0.101447416 |  |
| IFNG | 0.786354572 | | 2.195378727 | | 1.202081884 | | 4.009450455 | | | 0.010500286 |  |
| MCF2L-AS1 | -1.143043729 | | 0.318847059 | | 0.136865422 | | 0.742798624 | | | 0.008072195 |  |
| LINC00158 | -55.80436247 | | 5.81E-25 | | 1.33E-35 | | 2.55E-14 | | | 8.06E-06 |  |
| INE1 | -6.424599657 | | 0.001621182 | | 7.96E-05 | | 0.033033825 | | | 2.95E-05 |  |
| LINC00266-1 | -106.7641051 | | 4.29E-47 | | 2.37E-72 | | 7.78E-22 | | | 0.000320689 |  |
| EIF5A2 | 0.55665548 | | 1.744827122 | | 1.188180353 | | 2.562255535 | | | 0.004518554 |  |
| NR3C2 | -4.732821779 | | 0.0088016 | | 0.000440569 | | 0.175836429 | | | 0.001950895 |  |
| RBM26-AS1 | 11.47477047 | | 96256.37361 | | 215.3610573 | | 43022120.98 | | | 0.000228322 |  |
| FBN2 | 0.153298548 | | 1.165672937 | | 0.976774286 | | 1.391102751 | | | 0.089234554 |  |
| ITPKB-IT1 | -15.89398635 | | 1.25E-07 | | 8.45E-11 | | 0.000185251 | | | 1.98E-05 |  |
| ENDOU | 0.305648667 | | 1.357505286 | | 1.130929227 | | 1.629474735 | | | 0.001036003 |  |
| MMP11 | 0.045208592 | | 1.046246076 | | 1.022612263 | | 1.070426095 | | | 0.000105283 |  |
| TRIM71 | -140.3560561 | | 1.11E-61 | | 2.08E-90 | | 5.90E-33 | | | 3.20E-05 |  |
| ENPP4 | 1.848489265 | | 6.350218775 | | 2.662412019 | | 15.146145 | | | 3.07E-05 |  |
| GFI1 | -1.497990411 | | 0.223579011 | | 0.113402 | | 0.440799758 | | | 1.52E-05 |  |
| PLAU | 0.031005306 | | 1.031490977 | | 1.020238148 | | 1.042867919 | | | 3.03E-08 |  |
| SOX11 | -4.392464864 | | 0.012370201 | | 0.002545285 | | 0.060119742 | | | 5.18E-08 |  |
| LINC00028 | 16.78612171 | | 19503835.85 | | 1919.472989 | | 1.98179E+11 | | | 0.000362598 |  |
| RRAGD | -1.463472397 | | 0.231431257 | | 0.128669796 | | 0.416262622 | | | 1.03E-06 |  |
| SLC12A5 | 11.08654514 | | 65286.7995 | | 33.34676451 | | 127819482.7 | | | 0.004146317 |  |
| LINC00355 | 0.975114656 | | 2.6514712 | | 1.553619196 | | 4.525111134 | | | 0.000349593 |  |
| HCG11 | -2.198076623 | | 0.11101648 | | 0.049189509 | | 0.250554623 | | | 1.21E-07 |  |
| CRNDE | -0.386938406 | | 0.679132924 | | 0.461750676 | | 0.99885404 | | | 0.049322963 |  |
| LINC00520 | -2.250510405 | | 0.105345442 | | 0.047543424 | | 0.233421599 | | | 2.95E-08 |  |
| HOXC6 | -1.147432112 | | 0.317450902 | | 0.123379485 | | 0.816789557 | | | 0.017328213 |  |
| HAR1A | -12.61849508 | | 3.31E-06 | | 4.09E-08 | | 0.000268129 | | | 1.82E-08 |  |
| hsa-miR-206 | 0.000443153 | | 1.000443252 | | 1.000234501 | | 1.000652046 | | | 3.15E-05 |  |
| hsa-miR-125b-5p | -0.008299533 | | 0.991734813 | | 0.987795919 | | 0.995689414 | | | 4.36E-05 |  |
| hsa-miR-27a-3p | 0.000434885 | | 1.00043498 | | 0.999930803 | | 1.000939411 | | | 0.090854741 |  |
| hsa-miR-135a-5p | 1.524641826 | | 4.593498003 | | 2.253783065 | | 9.362136151 | | | 2.71E-05 |  |
| hsa-miR-23b-3p | 0.004347562 | | 1.004357026 | | 1.002695599 | | 1.006021207 | | | 2.65E-07 |  |
| hsa-miR-24-3p | 0.000429183 | | 1.000429276 | | 1.000117217 | | 1.000741431 | | | 0.007010689 |  |
| hsa-miR-193a-3p | -0.11133382 | | 0.89464005 | | 0.813289597 | | 0.984127698 | | | 0.022085274 |  |
| hsa-miR-107 | -0.081118163 | | 0.922084728 | | 0.890539083 | | 0.954747818 | | | 4.94E-06 |  |
| hsa-miR-129-5p | -0.051651767 | | 0.949659512 | | 0.903601418 | | 0.998065266 | | | 0.041718977 |  |
| hsa-miR-301b-3p | -0.995465446 | | 0.369551398 | | 0.233486292 | | 0.584909011 | | | 2.15E-05 |  |
| hsa-miR-363-3p | 0.107149879 | | 1.113101072 | | 1.072910824 | | 1.15479681 | | | 1.12E-08 |  |
