# Supplementary material for: Identification of lncRNA Signature of Tumor-Infiltrating T Lymphocytes With Potential Implications for Prognosis and Chemotherapy of Head and Neck Squamous Cell Carcinoma
Source: Front Pharmacol. 2022 Feb 15;12:795205. doi: 10.3389/fphar.2021.795205 (PMC8886158; doi:10.3389/fphar.2021.795205)
Supplement: Supplementary file 4 [file Table9.DOCX]

| Table S9. Multivariate cox regression analysis of CeRNA network genes in HNSCC patients (T3-T4) | | | | | |  |
| --- | --- | --- | --- | --- | --- | --- |
|  |  |  |  |  |  |  |
| Genes | Coef | HR | HR.95L | HR.95H | P value |  |
| ANKRD10-IT1 | 0.082277268 | 1.085756814 | 0.992786635 | 1.187433248 | 0.071631122 |  |
| NETO2 | 0.097778082 | 1.102718045 | 1.045350517 | 1.16323383 | 0.000334431 |  |
| STC2 | 0.038197295 | 1.03893619 | 1.012693918 | 1.065858486 | 0.003429768 |  |
| SALL4 | -1.599445026 | 0.202008596 | 0.096245617 | 0.423993053 | 2.36E-05 |  |
| FAM201A | -0.630460418 | 0.532346643 | 0.299741828 | 0.945456795 | 0.031448358 |  |
| WT1 | 0.662333197 | 1.939311856 | 0.991873456 | 3.791744252 | 0.052854025 |  |
| MDS2 | 5.561523626 | 260.2190109 | 2.729568044 | 24807.56389 | 0.016765536 |  |
| LIN28A | -16.76856613 | 5.22E-08 | 1.64E-14 | 0.166290314 | 0.028179615 |  |
| EN2 | -0.252108708 | 0.77716025 | 0.618723896 | 0.97616733 | 0.030209715 |  |
| LINC00158 | -7.244223188 | 0.000714289 | 2.16E-06 | 0.235829805 | 0.014357956 |  |
| EIF5A2 | 0.122300293 | 1.130093409 | 0.998797801 | 1.278648304 | 0.052273572 |  |
| LRRC2 | -0.244603826 | 0.783014687 | 0.580329344 | 1.056489744 | 0.109505594 |  |
| SORBS2 | 0.586147819 | 1.797052494 | 1.289176155 | 2.505008841 | 0.000542543 |  |
| LINC00028 | -4.329233177 | 0.013177649 | 0.000194537 | 0.892633453 | 0.044138848 |  |
| HCG11 | -0.245085941 | 0.782637275 | 0.655191775 | 0.934873007 | 0.006880436 |  |
| CRNDE | -0.104229393 | 0.901018586 | 0.801595181 | 1.012773669 | 0.080603471 |  |
| LINC00520 | 0.085609268 | 1.08938059 | 1.007447818 | 1.177976714 | 0.031875648 |  |
| HOXC6 | 0.673809237 | 1.961695671 | 1.304933231 | 2.949001383 | 0.001197026 |  |
| hsa-miR-206 | 8.35E-05 | 1.000083525 | 1.000027458 | 1.000139596 | 0.003501743 |  |
| hsa-miR-125b-5p | 0.000729941 | 1.000730208 | 1.000036631 | 1.001424265 | 0.039063559 |  |
| hsa-miR-212-3p | -0.030777993 | 0.969690827 | 0.941612219 | 0.998606732 | 0.040076169 |  |
| hsa-miR-17-5p | -0.000556498 | 0.999443657 | 0.99884415 | 1.000043523 | 0.069094701 |  |
| hsa-miR-23b-3p | -0.000218869 | 0.999781155 | 0.99956498 | 0.999997377 | 0.047285131 |  |
| hsa-miR-193a-3p | 0.027053882 | 1.027423161 | 0.999201273 | 1.05644216 | 0.056944516 |  |
| hsa-miR-20b-5p | 0.008096182 | 1.008129045 | 1.002586892 | 1.013701834 | 0.003995438 |  |
| hsa-miR-142-3p | -9.52E-05 | 0.999904849 | 0.999798197 | 1.000011513 | 0.080391849 |  |
