# Supplementary material for: Identification of lncRNA Signature of Tumor-Infiltrating T Lymphocytes With Potential Implications for Prognosis and Chemotherapy of Head and Neck Squamous Cell Carcinoma
Source: Front Pharmacol. 2022 Feb 15;12:795205. doi: 10.3389/fphar.2021.795205 (PMC8886158; doi:10.3389/fphar.2021.795205)
Supplement: Supplementary file 5 [file Table2.DOCX]

| Table S2. Multivariate cox regression analysis of CeRNA network genes in HNSCC patients >65 years old | | | | | |
| --- | --- | --- | --- | --- | --- |
|  |  |  |  |  |  |
| Genes | Coef | HR | HR.95L | HR.95H | P value |
| ANKRD10-IT1 | -0.177440628 | 0.837410716 | 0.705913214 | 0.993403572 | 0.041760384 |
| CADM2 | 7.987550813 | 2944.077525 | 0.297328406 | 29151578.9 | 0.088834574 |
| NETO2 | 0.099553785 | 1.104677885 | 0.997276915 | 1.223645319 | 0.056428532 |
| STC2 | 0.040576162 | 1.041410622 | 1.011489702 | 1.072216635 | 0.006371129 |
| SALL4 | -1.907783731 | 0.148408936 | 0.060619086 | 0.363337915 | 2.96E-05 |
| TDRG1 | 1.702948615 | 5.490111774 | 1.003338237 | 30.04104318 | 0.049552459 |
| WT1 | -1.058208476 | 0.347077051 | 0.130258671 | 0.92479432 | 0.034317173 |
| EN2 | -0.229827232 | 0.794670884 | 0.626458248 | 1.008050921 | 0.058240119 |
| HOXA10 | 0.354882547 | 1.426013154 | 1.133548731 | 1.793935682 | 0.00244261 |
| XIST | -0.2048578 | 0.814763168 | 0.654542838 | 1.014202556 | 0.066694372 |
| MCF2L-AS1 | 0.347004707 | 1.414823386 | 1.127211228 | 1.775820861 | 0.002765154 |
| MRVI1-AS1 | -1.771831656 | 0.170021283 | 0.025240988 | 1.145249802 | 0.068666512 |
| NWD1 | -1.098257738 | 0.333451538 | 0.113623995 | 0.97857788 | 0.045566507 |
| ITPKB-IT1 | -7.594197713 | 0.000503364 | 2.17E-07 | 1.169561137 | 0.054812813 |
| E2F7 | -0.2200922 | 0.802444809 | 0.660173789 | 0.975376004 | 0.027080539 |
| GFI1 | -0.220780264 | 0.801892865 | 0.673381948 | 0.954929323 | 0.013231601 |
| PLAU | 0.005067097 | 1.005079957 | 1.002443479 | 1.007723369 | 0.000156162 |
| RRAGD | -0.281108872 | 0.754946139 | 0.627840216 | 0.907784589 | 0.002803441 |
| SLC12A5 | -1.63768172 | 0.194430264 | 0.060355759 | 0.626338372 | 0.006072418 |
| LINC00355 | -0.691566707 | 0.500790862 | 0.276836775 | 0.90591825 | 0.02221527 |
| CRNDE | -0.110562251 | 0.895330593 | 0.772664369 | 1.037470994 | 0.141385238 |
| FGD5-AS1 | 0.095752901 | 1.100487101 | 1.03462913 | 1.170537177 | 0.002356349 |
| HOXC6 | 0.302214407 | 1.352851256 | 0.972781443 | 1.881415949 | 0.07249927 |
| hsa-miR-206 | 0.00016194 | 1.000161953 | 1.000054695 | 1.000269223 | 0.003081338 |
| hsa-miR-125b-5p | 0.002270872 | 1.002273452 | 1.001317185 | 1.003230633 | 3.12E-06 |
| hsa-miR-23b-3p | -0.000354618 | 0.999645444 | 0.999249538 | 1.000041508 | 0.079328745 |
| hsa-miR-193a-3p | -0.063925193 | 0.938075172 | 0.898984423 | 0.978865713 | 0.003244542 |
| hsa-miR-20b-5p | -0.010977743 | 0.989082293 | 0.974516949 | 1.003865333 | 0.146977249 |
| hsa-miR-301b-3p | 0.182623771 | 1.200362712 | 1.063151232 | 1.355282858 | 0.003190804 |
| hsa-miR-363-3p | 0.008597716 | 1.008634783 | 0.999798178 | 1.017549489 | 0.055491651 |
