# Supplementary material for: Identification of lncRNA Signature of Tumor-Infiltrating T Lymphocytes With Potential Implications for Prognosis and Chemotherapy of Head and Neck Squamous Cell Carcinoma
Source: Front Pharmacol. 2022 Feb 15;12:795205. doi: 10.3389/fphar.2021.795205 (PMC8886158; doi:10.3389/fphar.2021.795205)
Supplement: Supplementary file 6 [file Table5.DOCX]

| Table S5. Multivariate cox regression analysis of CeRNA network genes in male HNSCC patients | | | | | |  |
| --- | --- | --- | --- | --- | --- | --- |
|  |  |  |  |  |  |  |
| Genes | Coef | HR | HR.95L | HR.95H | P value |  |
| CADM2 | -3.426238167 | 0.032509004 | 0.00054504 | 1.939004253 | 0.100482096 |  |
| NETO2 | 0.06744616 | 1.069772662 | 1.00291871 | 1.141083058 | 0.040512771 |  |
| STC2 | 0.042492559 | 1.043408292 | 1.025524171 | 1.061604294 | 1.46E-06 |  |
| SALL4 | -0.534669992 | 0.585862597 | 0.341765874 | 1.004298584 | 0.051851405 |  |
| MMP11 | 0.003366856 | 1.003372531 | 1.00003522 | 1.006720978 | 0.047626795 |  |
| E2F7 | -0.128651701 | 0.879280165 | 0.800644373 | 0.96563922 | 0.007114305 |  |
| ENPP4 | 0.133667977 | 1.14301325 | 1.009426067 | 1.294279327 | 0.035038361 |  |
| GFI1 | -0.179462433 | 0.835719346 | 0.711303609 | 0.981896923 | 0.029102341 |  |
| PLAU | 0.003101721 | 1.003106537 | 1.001073604 | 1.005143597 | 0.002729729 |  |
| SOX11 | -0.347264033 | 0.706618733 | 0.471932196 | 1.058012228 | 0.091766644 |  |
| LINC00355 | 0.347292087 | 1.415230036 | 1.134497027 | 1.765430853 | 0.00207997 |  |
| LINC00520 | 0.075627245 | 1.07856046 | 1.003981065 | 1.158679887 | 0.038579655 |  |
| hsa-miR-206 | 0.000104708 | 1.000104714 | 1.000056999 | 1.000152431 | 1.70E-05 |  |
| hsa-miR-135a-5p | 0.146663336 | 1.157964052 | 1.021086347 | 1.31319036 | 0.022307947 |  |
| hsa-miR-125a-5p | -0.000725127 | 0.999275136 | 0.9984294 | 1.000121588 | 0.09324387 |  |
| hsa-miR-142-3p | -8.35E-05 | 0.999916482 | 0.999822642 | 1.00001033 | 0.081116411 |  |
| hsa-miR-363-3p | 0.004189636 | 1.004198425 | 1.002143153 | 1.006257911 | 6.12E-05 |  |
