# Supplementary material for: Identification of lncRNA Signature of Tumor-Infiltrating T Lymphocytes With Potential Implications for Prognosis and Chemotherapy of Head and Neck Squamous Cell Carcinoma
Source: Front Pharmacol. 2022 Feb 15;12:795205. doi: 10.3389/fphar.2021.795205 (PMC8886158; doi:10.3389/fphar.2021.795205)
Supplement: Supplementary file 7 [file Table3.DOCX]

| Table S3. Multivariate cox regression analysis of CeRNA network genes in HNSCC patients <=65 years old | | | | | |
| --- | --- | --- | --- | --- | --- |
|  |  |  |  |  |  |
| Genes | Coef | HR | HR.95L | HR.95H | P value |
| CADM2 | -3.300982017 | 0.036846965 | 0.000508718 | 2.668860869 | 0.130863054 |
| NETO2 | 0.088740131 | 1.092796636 | 1.029400861 | 1.160096647 | 0.003611079 |
| STC2 | 0.088740131 | 1.066859676 | 1.027924378 | 1.10726975 | 0.000645074 |
| SALL4 | -0.657767482 | 0.518006503 | 0.267607742 | 1.002701697 | 0.050943265 |
| FAM201A | -0.421487724 | 0.656070043 | 0.386420806 | 1.113883864 | 0.118612894 |
| LIN28A | -7.467517763 | 0.000571345 | 6.00E-08 | 5.444148812 | 0.110162166 |
| ELAVL2 | -0.185598807 | 0.830606762 | 0.68565448 | 1.006202996 | 0.057858706 |
| PDPN | -0.005768714 | 0.994247893 | 0.987489076 | 1.001052971 | 0.09740521 |
| XIST | 0.094422427 | 1.099023905 | 1.015179725 | 1.189792816 | 0.019697649 |
| NRIP3 | -0.108304539 | 0.897354275 | 0.806192284 | 0.998824612 | 0.04753737 |
| LINC00158 | -4.575149192 | 0.010304762 | 8.27E-05 | 1.284448965 | 0.063128188 |
| NR3C2 | -0.441164245 | 0.643287041 | 0.391528493 | 1.056930017 | 0.081612107 |
| TRIM71 | 13.67944727 | 872787.3973 | 44.28317226 | 17201970906 | 0.006702803 |
| ENPP4 | 0.230501467 | 1.259231315 | 1.073259889 | 1.477427342 | 0.004697013 |
| GFI1 | -0.165917341 | 0.847116253 | 0.679537965 | 1.056020388 | 0.140132813 |
| PLAU | 0.00297041 | 1.002974826 | 1.000298844 | 1.005657968 | 0.029318988 |
| LINC00355 | 0.394966951 | 1.484335134 | 1.191139287 | 1.849700379 | 0.000435099 |
| LINC00520 | 0.10042396 | 1.105639565 | 1.021991671 | 1.196133866 | 0.012352293 |
| hsa-miR-206 | 0.000105968 | 1.000105974 | 1.000058464 | 1.000153486 | 1.23E-05 |
| hsa-miR-24-3p | 8.46E-05 | 1.000084639 | 0.99999974 | 1.000169545 | 0.050706445 |
| hsa-miR-107 | -0.004295916 | 0.995713299 | 0.989815273 | 1.001646469 | 0.156414099 |
| hsa-miR-20b-5p | 0.005711389 | 1.00572773 | 1.002299391 | 1.009167796 | 0.001044439 |
| hsa-miR-301b-3p | -0.17498202 | 0.839472114 | 0.713072957 | 0.988276758 | 0.035588672 |
| SLC12A5 | -0.716197149 | 0.488606826 | 0.164174598 | 1.454163034 | 0.198067817 |
