# Supplementary material for: Identification of lncRNA Signature of Tumor-Infiltrating T Lymphocytes With Potential Implications for Prognosis and Chemotherapy of Head and Neck Squamous Cell Carcinoma
Source: Front Pharmacol. 2022 Feb 15;12:795205. doi: 10.3389/fphar.2021.795205 (PMC8886158; doi:10.3389/fphar.2021.795205)
Supplement: Supplementary file 8 [file Table4.DOCX]

| Table S4. Multivariate cox regression analysis of CeRNA network genes in female HNSCC patients | | | | | |  |
| --- | --- | --- | --- | --- | --- | --- |
|  |  |  |  |  |  |  |
| Genes | Coef | HR | HR.95L | HR.95H | P value |  |
| ANKRD10-IT1 | 0.199669641 | 1.220999324 | 1.007134764 | 1.480277914 | 0.042120566 |  |
| NETO2 | 0.471106422 | 1.601765442 | 1.377545045 | 1.862481768 | 9.19E-10 |  |
| STC2 | 0.11673578 | 1.123822454 | 0.993640824 | 1.271059801 | 0.063111631 |  |
| E2F1 | -0.171355157 | 0.842522293 | 0.749880096 | 0.946609755 | 0.00393709 |  |
| AMOT | 0.837425969 | 2.310412243 | 1.427898896 | 3.73836323 | 0.000647859 |  |
| FRY-AS1 | 43.6488936 | 9.04636E+18 | 484477825.1 | 1.69E+29 | 0.000297693 |  |
| MALAT1 | -0.12128368 | 0.885782645 | 0.840002406 | 0.934057913 | 7.48E-06 |  |
| MCM3AP-AS1 | -5.12960654 | 0.005918889 | 0.000162632 | 0.215414076 | 0.005156755 |  |
| TTTY10 | 1008.302139 | Inf | 1.00E+290 | Inf | 6.51E-09 |  |
| FAM201A | -5.023860127 | 0.006579082 | 0.000824339 | 0.052507928 | 2.13E-06 |  |
| TDRG1 | 0.280470123 | 1.323751992 | 1.085444027 | 1.614380192 | 0.005612654 |  |
| WT1 | 4.830585009 | 125.2842317 | 17.65674832 | 888.9597574 | 1.35E-06 |  |
| MDS2 | -33.48686747 | 2.86E-15 | 7.73E-23 | 1.06E-07 | 0.000165783 |  |
| ELAVL2 | 0.872179492 | 2.39211878 | 1.613095452 | 3.547361226 | 1.44E-05 |  |
| PDPN | -0.045837459 | 0.955197209 | 0.938230391 | 0.972470851 | 5.37E-07 |  |
| EIF5A2 | -0.306387254 | 0.736101506 | 0.549356986 | 0.986326634 | 0.040152172 |  |
| RBM26-AS1 | 2.331135795 | 10.2896218 | 0.619276504 | 170.9677601 | 0.104000332 |  |
| NWD1 | -3.217694616 | 0.040047276 | 0.00326416 | 0.491331443 | 0.011885554 |  |
| FBN2 | 0.106691328 | 1.112590775 | 1.036220719 | 1.194589349 | 0.003275521 |  |
| LRRC2 | -1.797330409 | 0.165740758 | 0.057503848 | 0.477707139 | 0.000875395 |  |
| ITPKB-IT1 | -37.93611591 | 3.35E-17 | 1.02E-28 | 1.10E-05 | 0.005048396 |  |
| MMP11 | -0.027304507 | 0.973064891 | 0.956731066 | 0.989677576 | 0.001570672 |  |
| TRIM71 | -74.76361361 | 3.39E-33 | 4.32E-56 | 2.67E-10 | 0.005443144 |  |
| SORBS2 | 2.288065818 | 9.855856214 | 3.732968216 | 26.02162571 | 3.85E-06 |  |
| GFI1 | 0.390234639 | 1.477327391 | 1.152973017 | 1.89292914 | 0.002032687 |  |
| PLAU | 0.014510108 | 1.014615891 | 1.009110915 | 1.020150897 | 1.72E-07 |  |
| SLC12A5 | 4.283695812 | 72.50792108 | 3.964437987 | 1326.139704 | 0.003866817 |  |
| LINC00355 | -0.436725969 | 0.646148472 | 0.362649328 | 1.151271532 | 0.138352101 |  |
| HCG11 | -0.798051777 | 0.45020521 | 0.283514879 | 0.714899805 | 0.000718558 |  |
| CRNDE | 0.865105359 | 2.375256327 | 1.633254796 | 3.454355459 | 5.98E-06 |  |
| FGD5-AS1 | -0.170611145 | 0.843149373 | 0.765877738 | 0.928217168 | 0.000503588 |  |
| LINC00520 | -0.185996612 | 0.830276408 | 0.685977607 | 1.00492918 | 0.056199658 |  |
| HOXC6 | 4.76221646 | 117.0049755 | 22.84216847 | 599.3373314 | 1.11E-08 |  |
| hsa-miR-206 | 0.000241907 | 1.000241936 | 1.000093216 | 1.000390678 | 0.001429641 |  |
| hsa-miR-212-3p | -0.112965633 | 0.893181356 | 0.835222348 | 0.955162342 | 0.000966537 |  |
| hsa-miR-135a-5p | -2.047333882 | 0.129078584 | 0.051716335 | 0.322166697 | 1.15E-05 |  |
| hsa-miR-17-5p | -0.00137963 | 0.998621321 | 0.997051401 | 1.000193714 | 0.085674605 |  |
| hsa-miR-24-3p | 0.001315016 | 1.001315881 | 1.00088721 | 1.001744737 | 1.75E-09 |  |
| hsa-miR-193a-3p | -0.205819837 | 0.813979712 | 0.742375081 | 0.892490857 | 1.18E-05 |  |
| hsa-miR-129-5p | -0.007284891 | 0.99274158 | 0.984254305 | 1.001302041 | 0.096324914 |  |
| hsa-miR-20b-5p | -0.037655638 | 0.963044519 | 0.942040259 | 0.984517103 | 0.000817328 |  |
| hsa-miR-142-3p | 0.000705828 | 1.000706077 | 1.000486746 | 1.000925457 | 2.77E-10 |  |
