# Supplementary material for: Identification of lncRNA Signature of Tumor-Infiltrating T Lymphocytes With Potential Implications for Prognosis and Chemotherapy of Head and Neck Squamous Cell Carcinoma
Source: Front Pharmacol. 2022 Feb 15;12:795205. doi: 10.3389/fphar.2021.795205 (PMC8886158; doi:10.3389/fphar.2021.795205)
Supplement: Supplementary file 9 [file Table6.DOCX]

| Table S6. Multivariate cox regression analysis of CeRNA network genes in HNSCC patients (G1-G2) | | | | | |  |
| --- | --- | --- | --- | --- | --- | --- |
|  |  |  |  |  |  |  |
| Genes | Coef | HR | HR.95L | HR.95H | P value |  |
| NETO2 | 0.107944352 | 1.113985752 | 1.047820993 | 1.184328493 | 0.000549896 |  |
| AMOT | 0.139419637 | 1.149606416 | 0.992256076 | 1.331909116 | 0.063389713 |  |
| EN2 | -0.198160553 | 0.820238151 | 0.66180871 | 1.016593789 | 0.070347351 |  |
| HOXA10 | 0.176705392 | 1.193279491 | 0.960139598 | 1.483030121 | 0.111111999 |  |
| ENO1-AS1 | 0.880758711 | 2.412729577 | 1.037674019 | 5.609915928 | 0.040769483 |  |
| CEP55 | -0.014869402 | 0.985240602 | 0.964693416 | 1.006225425 | 0.166722555 |  |
| XIST | 0.115061163 | 1.121942057 | 0.978457703 | 1.286467443 | 0.099344787 |  |
| MCF2L-AS1 | 0.189124382 | 1.20819122 | 1.011641469 | 1.44292822 | 0.036822098 |  |
| NR3C2 | -0.405874182 | 0.666394007 | 0.387243288 | 1.146775131 | 0.142792379 |  |
| LOXL2 | -0.02197457 | 0.978265112 | 0.962300645 | 0.994494427 | 0.008855155 |  |
| NWD1 | -0.956206042 | 0.384348325 | 0.180357131 | 0.81906179 | 0.01324891 |  |
| LRRC2 | -0.316115143 | 0.728975509 | 0.501004979 | 1.060678666 | 0.098515806 |  |
| ITPKB-IT1 | -3.905801921 | 0.02012481 | 0.000177105 | 2.286830099 | 0.105786142 |  |
| ENPP4 | 0.332648573 | 1.394657093 | 1.141086696 | 1.704575484 | 0.001157903 |  |
| PLAU | 0.002473727 | 1.00247679 | 1.00041187 | 1.004545972 | 0.018703777 |  |
| LINC00028 | -3.618559053 | 0.026821297 | 0.001032142 | 0.696979312 | 0.029468238 |  |
| SLC12A5 | -1.643380939 | 0.193325315 | 0.063519368 | 0.588398128 | 0.003805204 |  |
| LINC00355 | -0.437009647 | 0.6459652 | 0.446637388 | 0.934250134 | 0.020275516 |  |
| HCG11 | -0.190468163 | 0.826572073 | 0.718527358 | 0.950863436 | 0.007700687 |  |
| CRNDE | -0.151466822 | 0.859446396 | 0.760358207 | 0.971447537 | 0.015373946 |  |
| HOXC6 | 0.535701469 | 1.708646384 | 1.282966806 | 2.275563524 | 0.000247889 |  |
| hsa-miR-125b-5p | 0.000755787 | 1.000756072 | 0.999859313 | 1.001653636 | 0.098460871 |  |
| hsa-miR-135a-5p | 0.19157255 | 1.211152699 | 1.061784806 | 1.381533106 | 0.004335027 |  |
| hsa-miR-23b-3p | -0.000173835 | 0.99982618 | 0.999625953 | 1.000026447 | 0.088913126 |  |
| hsa-miR-24-3p | 0.000113537 | 1.000113544 | 1.000018256 | 1.000208841 | 0.019517952 |  |
| hsa-miR-20b-5p | -0.006640838 | 0.993381164 | 0.985100968 | 1.001730957 | 0.119946393 |  |
| hsa-miR-193a-3p | -0.022853827 | 0.977405344 | 0.951507052 | 1.00400854 | 0.095318576 |  |
